# P-830. Comparison of Oral Antibiotic Use and Hospital Readmission Among People Who Use Drugs with *Staphylococcus aureus* Bacteremia and Before Medically Advised Discharge During Two Time Periods

**DOI:** 10.1093/ofid/ofae631.1022

**Published:** 2025-01-29

**Authors:** Farzana Anam Karim, Monica K Sikka, Amber C Streifel, Luke Strnad, Ellie Sukerman

**Affiliations:** Oregon Health and Science University, portland, Oregon; Oregon Health and Science University, portland, Oregon; Oregon Health and Science University, portland, Oregon; Oregon Health and Science University, portland, Oregon; Oregon Health & Science University, Portland, Oregon

## Abstract

**Background:**

Recent studies suggest the benefit to salvage oral (PO) antibiotics to support people who use drugs (PWUD) with serious bacterial infections who leave before medically advised (BMA) prior to completing antibiotics. We compared the use of PO antibiotics in PWUD with *S. aureus* bacteremia (SAB) discharged BMA before versus after the implementation of a multidisciplinary discharge planning conference for PWUD requiring long-term antibiotics and publication of recent literature suggesting the benefit of PO antibiotics for patients leaving BMA.

**Figure:**
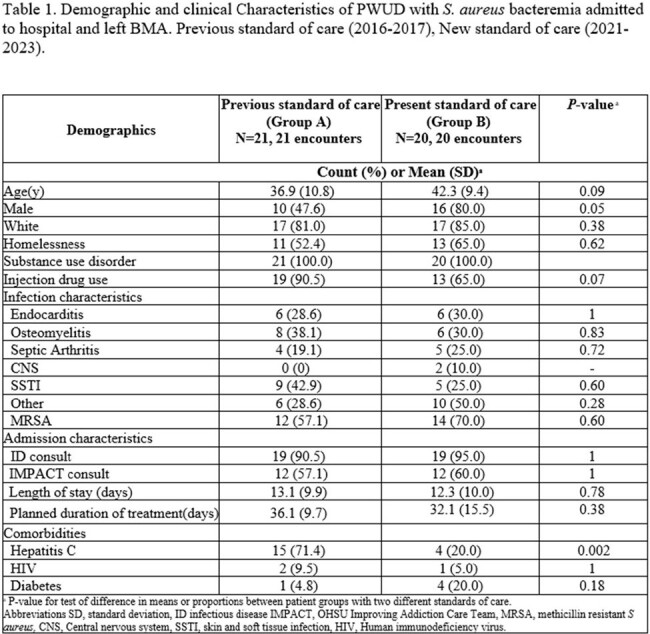

**Methods:**

We performed a retrospective chart review of PWUD with SAB admitted to a 500-bed teaching hospital over two time periods: 1/1/16 to 12/31/17 (group A) and 7/1/21 to 7/31/23 (group B). We compared 30 and 90-day readmission, PO and IV antibiotic recommendations, prescriptions, and percent of prescriptions dispensed.

**Figure:**
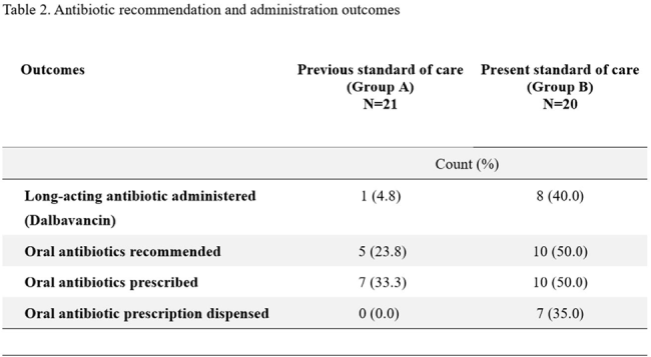

**Results:**

There were 21 patients in group A and 20 in group B. Univariable analysis showed no significant difference in the groups (Table 1). The incidence of 30 and 90-day readmission was similar (48% vs 50%, p=0.65) and (57% vs 65%, p=0.85), respectively. Long-acting antibiotics were administered on BMA discharge in 1 patient in group A and 8 in group B. PO antibiotic/s were prescribed on BMA discharge for 33% in group A and 50% in group B. While no patients in Group A filled their prescriptions, 35% in Group B did. When incorporating PO treatment that was dispensed and long-acting antibiotics administered, only 5% (1/21) in group A received treatment on BMA discharge compared to 75% (15/20) in group B.

**Conclusion:**

There was an improvement in providing recommendations and prescriptions for PO antibiotics as well as long-acting injectable antibiotics to provide treatment in situations of BMA discharge. These results suggest an impact of the publication of recent literature and our multidisciplinary discharge planning conference but still highlight room for improvement in recommending and prescribing PO antibiotics for PWUD with SAB who leave the hospital BMA. The lack of benefit in readmissions is likely due to the small sample size and limited duration of follow-up.

**Disclosures:**

**Monica K. Sikka, MD**, F2G: Grant/Research Support

